# Ophtalmologic changes in transthyretin familial amyloid polyneuropathy (ATTR-FAP)

**DOI:** 10.1186/1750-1172-10-S1-I9

**Published:** 2015-11-02

**Authors:** Natália Ferreira

**Affiliations:** 1 Ophthalmology Department, Centro Hospitalar do Porto, Hospital Santo António (HSA), Porto, Portugal

## Background

Familial amyloid polyneuropathy (FAP) is an inherited disorder with autosomal dominant transmission and multiple phenotypes, characterized by systemic accumulation of amyloid fibrils. The most common type of FAP is related to a mutant transthyretin (TTR). TTR is mainly synthesized in the liver, but few amount of TTR is produced in the eye, namely in retinal pigment epithelium, which explains the continuous intra-ocular amyloid deposition observed in patients submitted to liver transplantation. The incidence of ophthalmic manifestations related to FAP depends on mutation involved, geographical area of patient and time of evolution of the disease. More than 100 mutations of TTR have been described but the most frequent in Portugal is TTR Val30Met. Even the same population with the same mutation can present significant clinical variability. TTR Met30Val FAP patients have different phenotypes according to their age at onset of the disease.

## Methods

Dry eye, abnormal conjunctival vessels, pupillary abnormalities, vitreous opacities and glaucoma are common ocular changes associated to FAP. All are described as well as their etiology and incidence. Clinical cases with demographic data, TTR mutation involved, age at beginning of disease, period of evolution of disease, previous liver transplant or medical treatment, specific ophthalmologic alterations related to FAP and previous ocular surgeries are presented.

## Results

The most specific ocular manifestations of ATTR-FAP are deposits on lens anterior capsule and pupillary border, scalloped pupil and vitreous amyloidosis and the most severe one is glaucoma.

Amyloid deposits on anterior lens surface are central, disciform opacities with more dense border. Amyloid deposits on pupillary border are irregularities of white membranous material. Scalloped pupil, an irregular outlines and fringed edges of pupil, is pathognomonic of ATTR-FAP.

Peculiar vitreous opacities are the most common specific change of late onset TTR Met30Val population. There are four types of amyloid vitreous opacities: *pseudopodia lentis*, fibrils, spherical opacities and pre-vascular opacities. *Pseudopodia lentis* and typical fibrils, since numerous and dense, are also pathognomonic.

Dry eye is a common ocular change in FAP but a *non* specific. Signs of *keratoconjunctivitis sicca* like diminution of Break Up Time and *punctata* epitheliopathy are frequent and complications like corneal neovascularization or opacity and neurotrophic corneal ulcer are unusual.

Abnormal conjunctival vessels are a *non* specific modification of shape of vessels.

Pupillary reflex changes, light pupillary hiporreactivity or redilatation lag without loss of light reflex response, can be observed.

Glaucoma related to ATTR-FAP is an agressive secondary open angle glaucoma. Frequently, surgery is required (trabeculectomy or valve implants). Glaucoma leads to blindness if left untreated.

Amyloid retinal microangiopathy with peripheral retinal ischemia is a rare manifestation. It can be observed in advanced stage of disease.

## Conclusion

Frequently, the severity of ocular symptoms does not correlate with systemic symptoms, particularly in late onset disease. Vitreous opacities can be the first manifestation of the disease in older patients. Ocular manifestations are common in FAP TTR Val30Met patients and might be potentially severe with visual impairment like vitreous opacities and glaucoma. The most frequent specific alterations observed in early onset cases are signs related to dry eye and in late onset cases, vitreous opacities.

Vitrectomy is frequently required to remove amyloid in vitreous cavity to regain vision. In isolated cases with later onset and milder symptoms of the disease, vitreous opacities are frequently the first symptom. Ophthalmologist has an important role in follow-up of FAP patients to accurately treat sight-threatening manifestations and to diagnosis new cases, particularly in late onset TTR Met30Val.

